# The ADNP Derived Peptide, NAP Modulates the Tubulin Pool: Implication for Neurotrophic and Neuroprotective Activities

**DOI:** 10.1371/journal.pone.0051458

**Published:** 2012-12-14

**Authors:** Saar Oz, Yanina Ivashko-Pachima, Illana Gozes

**Affiliations:** 1 The Adams Super Center for Brain Studies, Tel Aviv University, Tel Aviv, Israel; 2 The Lily and Avraham Gildor Chair for the Investigation of Growth Factors, Tel Aviv University, Tel Aviv, Israel; 3 The Elton Laboratory for Neuroendocrinology, Department of Human Molecular Genetics and Biochemistry, Sackler Faculty of Medicine, Tel Aviv University, Tel Aviv, Israel; 4 Sagol School of Neuroscience, Tel Aviv University, Tel Aviv, Israel; Mental Health Research Institute of Victoria, Australia

## Abstract

Microtubules (MTs), key cytoskeletal elements in living cells, are critical for axonal transport, synaptic transmission, and maintenance of neuronal morphology. NAP (NAPVSIPQ) is a neuroprotective peptide derived from the essential activity-dependent neuroprotective protein (ADNP). In Alzheimer’s disease models, NAP protects against tauopathy and cognitive decline. Here, we show that NAP treatment significantly affected the alpha tubulin tyrosination cycle in the neuronal differentiation model, rat pheochromocytoma (PC12) and in rat cortical astrocytes. The effect on tubulin tyrosination/detyrosination was coupled to increased MT network area (measured in PC12 cells), which is directly related to neurite outgrowth. Tubulin beta3, a marker for neurite outgrowth/neuronal differentiation significantly increased after NAP treatment. In rat cortical neurons, NAP doubled the area of dynamic MT invasion (Tyr-tubulin) into the neuronal growth cone periphery. NAP was previously shown to protect against zinc-induced MT/neurite destruction and neuronal death, here, in PC12 cells, NAP treatment reversed zinc-decreased tau-tubulin-MT interaction and protected against death. NAP effects on the MT pool, coupled with increased tau engagement on compromised MTs imply an important role in neuronal plasticity, protecting against free tau accumulation leading to tauopathy. With tauopathy representing a major pathological hallmark in Alzheimer's disease and related disorders, the current findings provide a mechanistic basis for further development. NAP (davunetide) is in phase 2/3 clinical trial in progressive supranuclear palsy, a disease presenting MT deficiency and tau pathology.

## Introduction

The key cytoskeletal elements, microtubules (MTs), are essential for axonal transport and synaptic transmission. MTs are major regulatory targets for axonal regeneration and their stability and organization determine axonal fate [1]. The main building block of the MT tube is the α-β-tubulin heterodimer. Tubulin isotype microheterogeneity is expressed at the level of the single neuron, with pronounced developmental changes in β-tubulin associated with neurites [2], [3]. The isotype β3-tubulin is a neuronal marker in the developing and mature human nervous system [4], [5]. β3-tubulin plays a role in early neuritogenesis, concomitantly or in coordination with other MT associated proteins (MAPs) [6]. β3-tubulin may promote neurite extension by enhancing MT polymerization during early neuritogenesis [7].

The tyrosination/detyrosination cycle removes the C-terminal tyrosine of α-tubulin by a carboxypeptidase which is re-added by a tubulin-tyrosine-ligase (TTL). TTL is vital for neuronal organization and suppression of TTL in mice causes perinatal death [8]. Glu-tubulin is present in stable, long-living MT. In contrast, Tyr-tubulin is observed in newly formed MT [9]. Detyrosination is a consequence and not the cause of MT stabilization. Tyr-MT is predominantly located in the distal region of the axon, contiguous with the growth cone, and in cell bodies but is present in relatively lower amounts in the axonal shaft, where Glu-MT is the more dominant form [10]. In dendrites where MT display mixed polarity, Tyr-MT is more prominent than Glu-MT [11]. The tyrosination/detyrosination cycle is also implicated in interaction with MAPs and motor proteins leading to assembly and disassembly of MTs [12]. All isoforms of tau have been shown to bind Tyr-MT [13]. In this respect, tau pathology (tauopathy) is a major hallmark of Alzheimer’s disease and related neurodegenerative diseases such as progressive supranuclear palsy, PSP [14].

Activity-dependent neuroprotective protein (ADNP) is vital for brain formation in vivo [15], [16], and neurite outgrowth in vitro [17]. ADNP-haploinsufficiency results in tauopathy [18]. NAP (NAPVSIPQ), a neuroprotective peptide derived from ADNP [19], protects against ADNP deficiency [18]. Original data suggested that NAP protects neurons through MT reorganization [20], [21], promoting neurite outgrowth [22] and providing neuroprotection. The current study was aimed at testing the hypothesis that the mechanism of action of NAP involves 1] short-term changes in MT stability as indicated by changes in the α-tubulin tyrosination cycle (2 hrs.); 2] increase in MT content and protection against MT disruption and tau loss from MT in compromised cells (4 hrs.); 3] increase in MT-network area in the cell (24 hrs.); 4] increase in dynamic MT in the growth cone and 5] induction of neuron-like differentiation at the level of β3-tubulin expression (8 days). Our results indicate an effect of NAP on the MT pool toward differentiation/plasticity and protection of the MT-tau interaction in the face of toxicity.

## Materials and Methods

### NAP Treatment

NAP was provided by Allon Therapeutics Inc., Vancouver BC, Canada. The peptide was dissolved in double distilled H_2_O.

### Buffers

A series of solutions used is listed below. 1] SDS-polyacrylamide gel electrophoresis sample buffer (45% glycerol, 20% b-mercaptoethanol, 9.2% SDS, 0.04% bromophenol blue, 0.3 M Tris-HCl, pH 6.8). 2] MT-buffer (80 mM PIPES pH 6.8, 1 mM MgCl_2_, 2 mM EGTA, 5% Glycerol, with or w/o 0.5% TritonX-100). 3] TBS-T (20 mM Tris pH 7.5, 136.8 mM NaCl, and 0.05% v/v Tween 20). 4] Modified RIPA lysis buffer (50 mM Tris-HCL pH 7.4, 150 mM NaCl, 2 mM EGTA, 1% Triton X-100, 0.1% SDS, 0.1% sodium Deoxycholate).

### Antibodies and Cell Labeling Dyes

A series of antibodies used is listed below. Monoclonal anti-β3-tubulin antibody (T8578, Sigma, Rehovot, Israel), monoclonal anti Tyr-α-tubulin antibody (YL1/2) (VMA1864, Abcys, Paris, France), polyclonal anti Glu-α-tubulin antibody (L4) (AbC0101, Abcys, Paris, France), monoclonal anti-tau (tau-5) (ab80579, Abcam, MA, USA), monoclonal anti-tau (tau-5) (Biosource International, Camarillo, CA, USA), monoclonal anti-total tau (AT-5004, MBL, Billerica, MA, USA), monoclonal anti Actin (ab1801, Abcam, MA, USA), monoclonal anti α-tubulin (DM1A) (T6199, Sigma, Rehovot, Israel). The secondary antibodies used were Peroxidase AffiniPure Goat anti–mouse (Jackson ImmunoResearch, Suffolk, UK), Cy3-conjugated Goat Anti-Rat IgG, Cy5- conjugated goat anti-rabbit IgG, Rhodamine Red-X-AffiniPure Fab Fragment Goat Anti-Rabbit IgG (Jackson ImmunoResearch). DyLight 488-labeled secondary goat anti-mouse IgG, DyLight 633-labeled secondary goat anti-rabbit IgG (KPL, Gaithersburg, MD, USA). Additional compounds used for microscopy included, coumarin Phalloidin (P2495, Sigma-Aldrich) which identifies actin filaments and sulfonated DiI: DiIC18(5)-DS (D12730, Invitrogen, NY, USA). The latter is a lipophilic tracer: long-chain dialkylcarbocyanine that uniformly labels neurons via lateral diffusion in the plasma membrane.

### Cell Lines

NIH3T3 cells, mouse fibroblasts (ATCC, Bethesda, MD, USA) were grown in DMEM supplemented with 10% fetal calf serum, 2 mm glutamine, 100 U/ml penicillin and 100 mg/ml streptomycin. Rat pheochromocytoma cells (PC12, ATCC, Bethesda, MD, USA) were seeded at 3×10^4^ cells/cm^2^ on poly-L-Lysine coated plastic tissue culture dishes (Corning, Lowell, MA, USA) to form an adherent monolayer. Cells were maintained in Dulbecco’s modified Eagle’s medium (DMEM) supplemented with 10% horse serum, 5% fetal calf serum, 2 mM glutamine, 100 U/ml penicillin and 100 mg/ml streptomycin (Biological Industries, Beit Haemek, Israel). When indicated, PC12 differentiation was induced by nerve growth factor (NGF-β, N2513, Sigma), at concentrations of 50 ng/ml, by replacing half of the medium every other day. The cells were incubated in 95% air/5% CO_2_ in a humidified incubator at 37°C.

### Primary Cell Cultures

Rat (Sprague–Dawley) cortical astrocytes from newborn pups were prepared as before [19], [21]. For neuronal enriched cultures, newborn rats were sacrificed at postnatal day 1. Cerebral cortex tissue was then dissected and dissociated individually from each pup with the Papain Dissociation System (PDS), (Worthington Biochemical Corporation) according to the manufacturer’s instructions. Cortical neurons were maintained in neurobasal medium (NB), (Gibco) supplemented with NeuroCult B27-SM1 (STEMCELL), 1% Glutamax (Gibco), and grown on poly-D-lysine-coated cell culture glass cover slips for 13 days. The cells were incubated in 5% CO_2_ in a humidified incubator at 37°C.

### Cell Lysate Preparation

Cells were washed with ice-cold PBS and lysed on ice for 30 min in modified RIPA lysis buffer with protease inhibitors cocktail (Sigma), and subjected to centrifugation at 14,000 g for 15 min. Protein concentrations in the supernatant were determined by the BCA method (Pierce, Rockford, IL, USA).

### Immunoblotting

Proteins were separated by electrophoresis on a 10% polyacrylamide gel or 4–20% precast iGels (NuSep, Bogart, GA, USA), electrotransfered to nitrocellulose membranes (Millipore, Billerica, MA, USA), and probed with antibodies. Secondary antibodies were visualized with a chemiluminescence kit (Pierce). The densitometric analysis of western blots was performed with MiniBIS Pro Gel imaging system and software (DNR, Maale HaHamisha, Israel).

### Polymerized vs. Soluble Tubulin Separation Assay

To quantify tubulin polymerization, a simple assay was developed by modifying a method originally described [23], [24]. Cells grown to confluence in 6-well plates were washed with MT-buffer w/o TritonX-100 and lysed at 37°C for 5 min, with 150 µl of MT-buffer with TritonX-100 in order to extract soluble (cytosolic) tubulin (*S*); Pelleted cells were rinsed once again with equal volume of modified RIPA buffer in order to collect the polymerized (cytoskeletal) tubulin (*P*). The cytosolic and cytoskeletal fractions were each mixed with sample buffer and heated at 95°C for 5 min. An equal volume of each fraction was analyzed by immunoblotting, and the results following ECL development were quantified by densitometry. The percentage of polymerized tubulin was determined by dividing the densitometry value of polymerized tubulin by the total tubulin content (the sum of the densitometry values of soluble and polymerized tubulin).

### Microplate Reader

#### “In cell western”

The assay was performed as previously described [25]. Briefly, cells were permeabilized for 5 minutes prior to fixation in order to remove soluble tubulin. Cells were then fixed and incubated with tubulin antibodies YL1/2 and L4 at a 1∶4000 dilution, followed by secondary antibodies and Hoechst staining. Fluorescence was measured using the Tecan Microplate fluorescent reader (Neoteck scientific instrumentation, Switzerland) infinite F200 model, Magellan software version 6.3. The fluorescent signal was normalized to the corresponding Hoechst signal in each well which gives an indication of the relative amount of cells in each well.

### Immunostaining

Cultured cells plated on glass coverslips were fixed and permeabilized simultaneously, with 3% paraformaldehyde, 0.075% glutaraldehyde (Fluka Biochemika) in MT-buffer with TritonX-100, for 10 min, quenched with 1 mg/ml NaBH_4_ in PBS, blocked with 2% BSA and 5% normal goat serum in TBS-T, and incubated with primary antibodies followed by the appropriate secondary antibodies. Nuclei were visualized with Hoechst dye.

### Confocal Microscopy and Image Analysis

Images were collected with a Leica SP5 confocal laser scanning microscope (CLSM, Mannheim, Germany) with 63X oil immersion optics, laser lines at 488, 561, 633 nm or with LSM 510 META (Zeiss, Jena, Germany) confocal laser scanning microscope with 63X oil immersion optics, laser lines at 488, 568, 633 nm. When comparing fluorescence intensities, identical CLSM parameters (e.g., scanning line, laser light, gain, and offset etc.) were used. All of the fluorescent signals acquired were above the autofluorescent background as measured from a control slide stained with secondary antibody without a primary antibody. To compare the relative levels of protein expression, we used the average integrated density image (AID) analysis procedure for cell immunostains. In brief, integrated density is defined by the sum of the values of the pixels in the selected region of interest (ROI). This is equivalent to the product of Area and Mean Gray Value. AID for the positive stained area was determined by measuring the fluorescent intensity of the ROI, which is above the positive cut-off intensity. Positive cut-off intensities were determined based on the fluorescence intensities histogram for each antibody staining. For measurements of the MT containing area in a given cell, the chosen focal plane was the one showing the maximal area on the z-axis (focal axis). Analysis was performed using the MICA software (Cytoview, Petach Tikva, Israel) and ImageJ (NIH, Bethesda, MD, USA).

### Zinc toxicity

On the day of the experiment, the growth medium was aspirated and fresh medium containing ZnCl_2_ (400 µM; Sigma, Rehovot, Israel) with or without NAP was added to the cells. The cells were incubated for 4 hrs. Survival was measured using the MTS assay (CellTiter 96 AQueous Non-Radioactive Cell Proliferation Assay; Promega, Madison, WI, USA) according to the manufacturer’s instructions and read in an ELISA plate reader at 490 nm. The absorbance at 490 nm of the control untreated group was used as reference and calculated to be 100%.

### Statistical Analysis

Data are presented as the mean ± SEM from at least 3 independent experiments performed in triplicates in western blot analysis and at least 3 independent experiments in duplicates or more in confocal studies. Statistical analysis of the data was performed by using one-way ANOVA with Dunnett’s post-test using GraphPad Prism version 5.00 (GraphPad Software, San Diego California USA, www.graphpad.com), * p<0.05, ** p<0.01, *** p<0.001. All Pairwise Multiple Comparison Procedures (Student-Newman-Keuls Method) were performed using SigmaStat.

## Results

### NAP Effects on the Tubulin Tyrosination Cycle

**Figure 1:**
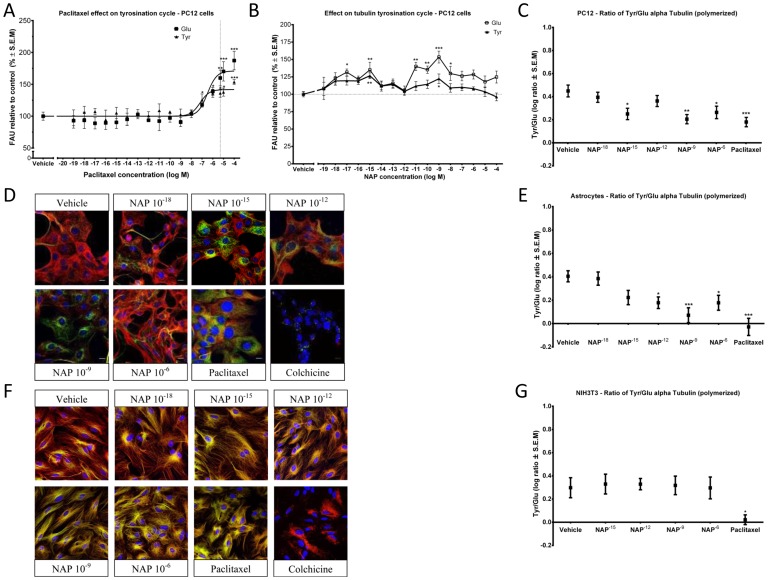
NAP affects the α-tubulin tyrosination cycle. Glu-MT and Tyr-MT fluorescence levels in cultured cells after exposure for 2 hrs to different drug concentrations. Cells were permeabilized during fixation to remove non-polymerized tubulin subunits, therefore only tubulin incorporated into MT was assessed. (**a**) Concentration response analysis of fluorescence intensity units (FAU) normalized to Hoechst signal in paclitaxel-treated PC12 cells evaluated by “In cell western”, microplate reader method (Glu-MT, ANOVA, p<0.0001, n = 9 per treatment), (Tyr-MT, ANOVA, p<0.0001, n = 9 per treatment). The dotted line indicates the paclitaxel concentration, 5 µM, used in the following experiments as a control. (**b**) Concentration-response analysis of fluorescence intensity units (FAU) normalized to Hoechst signal in NAP- (10^−18^M - 10^−6^M) or vehicle-treated cells. PC12 cells were evaluated by “In cell western”, microplate reader method. (Glu-MT, ANOVA, p<0.0001, n = 9 per treatment), (Tyr-MT, ANOVA, p = 0.0139, n = 9 per treatment) (**c**) Concentration response analysis of fluorescence intensity ratio levels (Tyr-MT/Glu-MT) in PC12 cells treated with NAP (10^−18^M - 10^−6^M) or vehicle, evaluated by image analysis of confocal images. The control compound was paclitaxel, 5 µM, (ANOVA, p = 0.0001, n>34 cells per treatment). The fluorescence intensities of both Tyr-MT and Glu-MT in the colchicine-treated cells were below the threshold level of measurements (see d). (**d**) Comparison of the effect on Glu-MT and Tyr-MT and integrity of the MT network. Control compounds were paclitaxel, 5 µM and colchicine, 2 µM (bottom row). PC12 stained for Tyr-MT (red), Glu-MT (green), Hoechst (blue). Bars: 10 µm. (**e**) Concentration response analysis of fluorescence intensity ratio levels (Tyr-MT/Glu-MT) in rat cerebral cortical astrocytes evaluated by image analysis of confocal images. (ANOVA, p<0.0001, n = 27). The fluorescence intensities of both Tyr-MT and Glu-MT in the colchicine-treated cells were below the threshold level of measurements (see f). (**f**) Comparison of the effect on Glu-MT and Tyr-MT and integrity of the microtubular network. Control compounds were paclitaxel, 5 µM and colchicine, 2 µM (bottom row). Rat cerebral cortical astrocytes stained for Tyr-MT (red), Glu-MT (green), Hoechst (blue). Bars: 10 µm. (**g**) Concentration response analysis of fluorescence intensity ratio levels (Tyr-MT/Glu-MT) in NIH3T3 cells evaluated by image analysis of confocal images. (ANOVA, p = 0.9966, n = 18).

To evaluate the effects of NAP on the tubulin tyrosination cycle, we conducted two assays: “In cell western” based on the method previously described [25], (Fig. 1a,b), and confocal image analysis (Fig. 1c–g). In both assays, polymerized MTs in the cells were evaluated for immunoreactive Glu-α-tubulin and Tyr-α-tubulin. As references, we used two drugs known to exhibit a MT-depolymerizing effect and a MT-stabilizing effect, colchicine and paclitaxel, respectively. PC12 cells (neuronal-like model cells) were incubated (2 hrs.) in the presence of drugs. Results showed that at concentrations of 10^−19^M - 10^−8^M paclitaxel did not have a significant effect. At concentrations >10^−8^M paclitaxel treatment had a significant effect on the tyrosination cycle increasing both Glu-MT and Tyr-MT (Fig. 1a), with a highly significant effect on the stable Glu-MT at concentrations >10^−7^M as expected [25], (Fig. 1a, the dashed line in the figure signifies a concentration of 5 µM paclitaxel, which was used in all subsequent experiments). NAP showed a concentration-dependent effect on the tyrosination cycle of α-tubulin, increasing the levels of Glu-MT and also the levels of Tyr-MT, although to a much lesser extent compared to Glu-MT. Two peaks of activity were seen at 10^−15^M and 10^−9^M NAP (Fig. 1b-d). Fig. 1c shows the results of the confocal quantitative analysis with ratios of Tyr-MT/Glu-MT, indicating ratios significantly different from control at 10^−15^M, 10^−9^M and 10^−6^M NAP and suggesting an increase in stabilized polymerized MT after NAP treatment at these concentrations. Colchicine, a MT-destabilizing agent, significantly reduced the fluorescence intensities of both Tyr-MT and Glu-MT, below the threshold level of detection, as defined in the method section (Fig 1d). Rat cerebrocortical astrocyte cell cultures treated with NAP showed similar increases in stabilized polymerized MT with Tyr-MT/Glu-MT ratios significantly different from control at 10^−12^M - 10^−6^M NAP. Treatment of astrocytes with paclitaxel and colchicine showed similar results as in PC12 cells (Fig. 1e,f). To validate these results, we conducted the same experiment using NIH3T3 fibroblasts. Paclitaxel showed the same effect as was observed in PC12 cells, while NAP had no effect on the ratios of Tyr-MT/Glu-MT in fibroblasts (Fig. 1g), suggesting cellular specificity.

### NAP Effect on Polymerized vs. Soluble Tubulin in PC12 Cells

**Figure 2:**
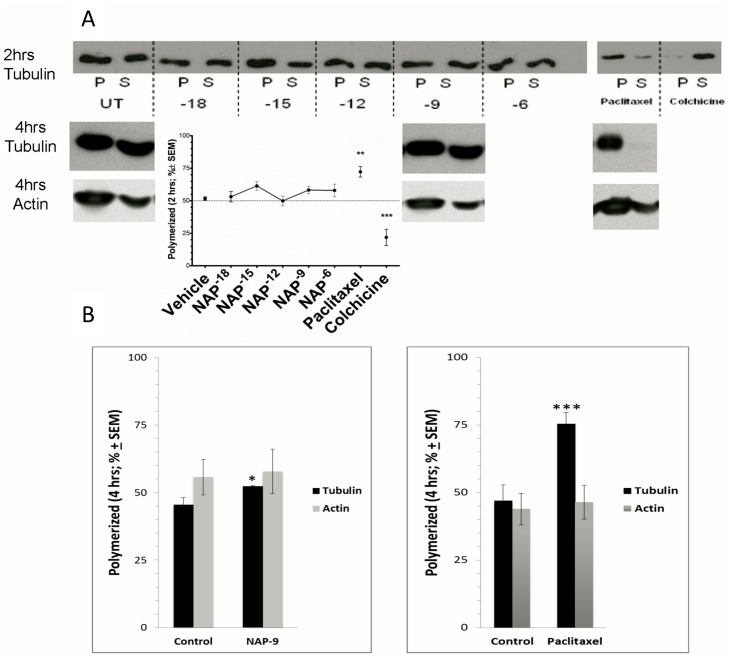
NAP effect on the polymerized tubulin fraction in PC12 cells. PC12 cells were treated for 2 hrs with either NAP (10^−18^M - 10^−6^M) or vehicle, and control compounds paclitaxel, 5 µM and colchicine, 2 µM. An additional time point (4 hrs.) was included as well for NAP (10^−9^M), vehicle and paclitaxel, 5 µM. (**a**) Cell lysates were separated into polymerized (*P*) or soluble (*S*) protein fractions. Aliquots of equal volume for each pair were resolved on adjacent lanes by SDS polyacrylamide gel electrophoresis, the blot probed with anti α-tubulin (2 hrs.) and with anti actin. The intensity of each band was quantified by densitometry and the percentage of polymerized MT was calculated by dividing the densitometric value of polymerized tubulin (*P*) by the total tubulin content (the sum of *P* plus *S*) in the 2 hr-incubation period and plotted as a line graph (ANOVA, p = 0.1868, n = 7). (**b**) The 4 hr-incubation samples shown in (**a**) were subjected to densitometry as above and the data plotted in bar graphs. Pairs of treatment were compared for each of the variables tubulin, actin (t-test p<0.05; ***p<0.005, n = 3).

To further analyze the effects of NAP on MT, we examined the relative levels of polymerized and soluble tubulin pools in PC12 cells after drug treatment. Cells were treated for 2 hrs. with increasing NAP concentrations and control compounds or treated with vehicle and lysed as described in the methods section and the levels of polymerized and soluble tubulin were assessed by western blot analysis (Fig. 2a, pictures) followed by densitometric evaluations (Fig. 2a, graph). In the vehicle-treated cells, about 50% of the tubulin was found in soluble form *(S)* or the polymerized *(P)* fraction. As expected, paclitaxel-treated cells showed a high level of polymerized *(P)* tubulin of up to 80% of the total tubulin pool, while colchicine reduced the levels of polymerized *(P)* tubulin to 20%. NAP treatment did not result in a statistically significant change in the free tubulin to MT balance in the cells, although a trend was observed.

To assess whether an extended time period of incubation with NAP will increase MT polymerization, we have extended the incubation time with NAP to a period of 4 hrs. Under these conditions, NAP (10^−9^M) modestly, but significantly increased the content of tubulin (∼1.15-fold) in polymerized MT (Fig. 2a and b). Paclitaxel (5 µM), tested under the same conditions (incubation for 4 hrs.), also increased the relative tubulin content in the MT fraction, similar to the results described above for the shorter incubation period (Fig. 2a and b).

### NAP Effect on Zinc Intoxication and on Tau-tubulin Interaction

**Figure 3:**
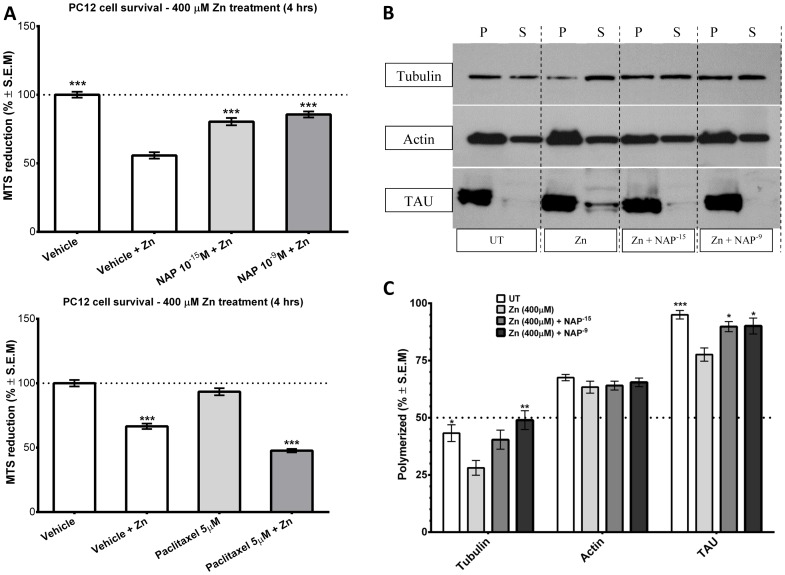
NAP enhances tau-microtubule interactions and microtubule polymerization under zinc intoxication. (**a**) Zinc exposure (400 µM) resulted in PC12 cell death which was protected against by NAP treatment. Results of mitochondrial activity (MTS cell viability) are shown –100– is 100% survival – control w/o zinc treatment. (ANOVA, ***p<0.0001, n = 18/group, post hoc comparison were made in reference to vehicle+Zn treatment), upper panel. The lower panel shows a similar experiment with 5 µM paclitaxel (ANOVA, ***p<0.0001, n = 10/group, post hoc comparisons made in reference to vehicle, or to vehicle+Zn treatment). (**b**) Cell lysates were separated into polymerized (*P*) or soluble (*S*) protein fractions as in Figure 2. Aliquots of equal volumes for each pair were separated on adjacent lanes by SDS polyacrylamide gel electrophoresis, the blot was probed with antibodies recognizing α-tubulin, or total tau or actin and the percentage of the polymerized fraction was calculated for each ‘*P*’ and ‘*S*’ pair. (**c**) The intensity of each band was quantified by densitometry, and the percentage of the polymerized fraction was determined. (Actin, ANOVA, p = 0.4774, n = 6; Tubulin, ANOVA, p = 0.007, n = 6; TAU, ANOVA, p = 0.0022, n = 5, post hoc determinations were against the zinc-treated group for each of the variables).

Previous studies have shown NAP protection against zinc intoxication in neurons [20] and in glial cells [21]. Here, we exposed PC12 cells to increasing zinc concentration for 4 hrs. Significant cell death was observed at zinc concentration >200 µM (data not shown), corroborating previously published data that established the EC50 for zinc cell killing effect at 308±38 µM [26]. We chose to work with zinc concentrations that gave consistent and significant cell death and hence we worked with 400 µM zinc. NAP treatment at concentrations of 10^−15^M and 10^−9^M showed a significant protection effect against cell death induced by zinc toxicity (Fig. 3a, top panel).

The control compound, paclitaxel (5 µM), while not affecting cell viability when tested on its own, when added in combination with Zn, enhanced cell death (Fig. 3b, bottom panel).

Further evaluation of the MT pool in the presence of 400 µM zinc, showed about 40% reduction in the polymerized tubulin content as compared to non-compromised cells. There was no effect on the actin-microfilament pool, suggesting that zinc had selective effects on the MT pool (Fig. 3b, western analysis, Fig. 3c, densitometric quantification). NAP treatment (10^−15^M and 10^−9^M) restored the polymerized MT pool to control levels. As previous studies indirectly suggested 1] increased tau association with the MT pool and inhibition of tauopathy in the presence of NAP [27], and 2] decreased tau association with the MT pool in the presence of high zinc concentrations [28], we further tested whether zinc intoxication and MT loss in our model system was accompanied by decreased tau-MT association and possible amelioration by NAP. Results showed that in untreated cells almost all the immunoreactive tau was associated with the polymerized MT fraction. Zinc intoxication decreased tau association with MT by ∼25% and this decrease was completely reversed following NAP treatment (Fig. 3b and c).

### NAP Effect on Total Polymerized α-tubulin (MT Network Area/Cell)

**Figure 4:**
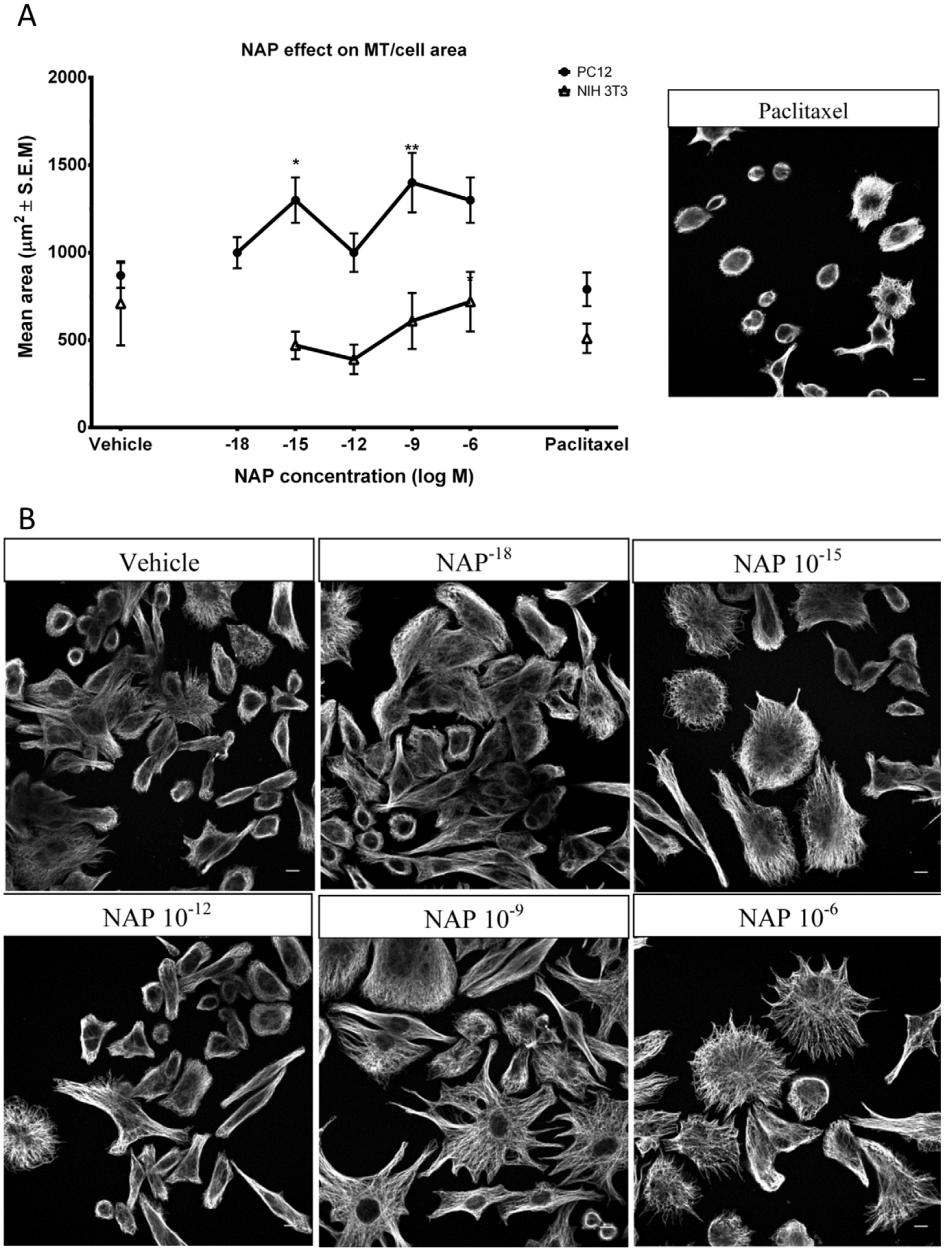
Treatment with NAP increases total polymerized α-tubulin network area in PC12 cells. (**a**) Concentration response analysis of MT network area/cell as determined by the outer boundaries of fluorescently probed MT (monoclonal anti α-tubulin) in PC12 or NIH3T3 cells, evaluated by image analysis of confocal images. Cultured cells were exposed for 24 hr. to different concentrations of NAP (10^−18^M - 10^−6^M) and compared to vehicle-treated cells and paclitaxel, 5 µM treated cells. (PC12 - ANOVA, p = 0.0081, n = 87 cells per treatment; NIH3T3 - ANOVA, p = 0.3892, n = 90). Left side, Representative field-images of PC12 cells treated with paclitaxel with fluorescently probed MT. Bar: 10 µm (**b**) Representative field-images of PC12 cells with fluorescently probed MT (control and NAP-treated as indicated). Bars: 10 µm.

To further analyze the effects of NAP on MT and in consideration of the α-tubulin tyrosination cycle coupled with the results of polymerized vs. soluble tubulin, we evaluated the effect of NAP on the total polymerized α-tubulin after 24 hr. treatment. In order to do so, we assessed total polymerized α-tubulin area as defined by the outer boundaries of the MT network probed with antibodies against total α-tubulin, as an indication for the whole polymerized α-tubulin pool. PC12 cells were treated with NAP or vehicle for 24 hrs. The MT network area/cell was determined by confocal image analysis. A concentration-dependent increase in mean the MT network area/cell in response to NAP treatment was observed at NAP concentrations of 10^−15^M and 10^−9^M and a lesser effect was seen at 10^−12^M (Fig. 4a), similar to the concentration-dependent curve observed in the tyrosination cycle (Fig. 1). In contrast, no effect was observed on NIH 3T3 cells, extending and corroborating the finding on the tyrosination cycle. All measurements were relative to vehicle-treated cells. In contrast to NAP, a 24 hr. paclitaxel treatment insignificantly reduced the area covered by the alpha tubulin MT network exhibiting a highly condensed and bundled MT network compared to a widely spread network after NAP treatment (Fig. 4a, left side quantification, right side picture, compared to Fig. 4b).

### NAP Affects MT Invasions into Growth Cones in Primary Neuronal Cells

**Figure 5:**
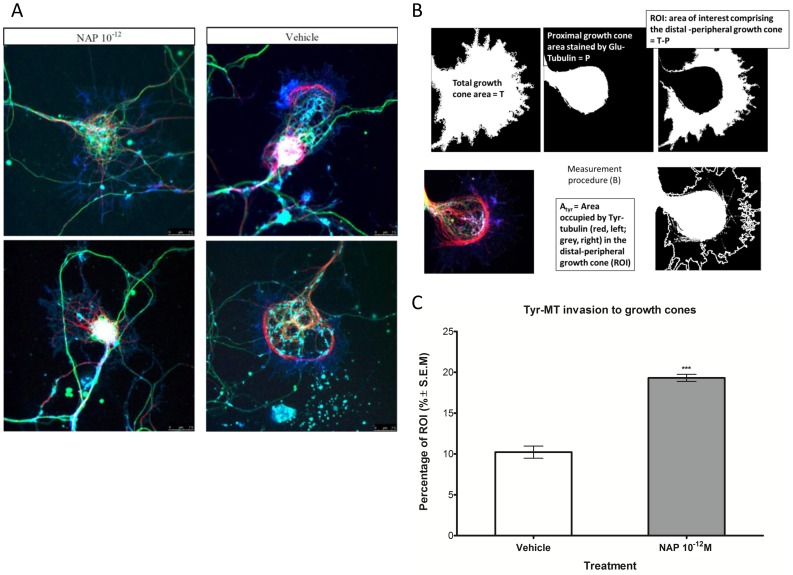
NAP effect on Tyr-MT invasion to growth cones. (a) Glu-MTs (green) do not invade the peripheral domain of the growth cone. NAP clearly affected Tyr-MT (red) invasion to the peripheral domain of the growth cone in terms of number and length of this invasions. Actin is labeled in blue (Coumarin Phalloidin) and cell membranes are stained in cyan (Dil). Bars: 7.5 µm. (b) Analysis of MT invasion - the total area in the image covered by actin and membrane dyed using DiI determined the growth cone area (T). From this determined area we subtracted the area occupied by Glu-MT (proximal growth cone area, P) which gave us our region of interest (ROI), as illustrated, with a representative picture and termed A_tyr_ (area including Tyr-MT). (c). The percentage of the ROI penetrated by Tyr-MT was obtained, calculated and graphed, right hand side, (t-test, p<0.0001, n = 22/treatment group).

Primary neurons show extensive, cell site specific, posttranslational modifications (PTMs) of MT [29], [30], making those MT species insensitive to the subtle changes observed by the “field of cells = multiple cell” analysis we used in Fig. 1. In primary neurons, a major change that accompanies the transformation of a minor neurite into the future axon occurs at the growth cone. It involves an increase in growth cone size, the expansion of the peripheral lamellipodial veil, a shortening of actin ribs, increased actin dynamics, an increase in the number and length of recently assembled dynamic MTs, and penetration of MTs into the central and peripheral domains of the growth cone [31], [32], [33]. Few Tyr-MTs penetrate into the peripheral regions of growth cones, which generally do not contain a MT network [34], [35], [36]. Here, using a previously described image analysis technique, we quantified the area of the growth cone invaded by MTs in cultured neurons [37]. We probed with Glu-MTs together with actin and Dil staining of the membrane in order to determine the growth cone area and to separate the three regions of the growth cone: the central domain, the transitional domain, and the peripheral domain, the latter being our region of interest (ROI). The central domain is located in the center of the growth cone nearest to the axon. This region, composed primarily of a MT-based cytoskeleton, is generally thicker, while containing many organelles and vesicles. The transitional domain is the region located in the thin band between the central and the peripheral domains. The peripheral domain is the thin region surrounding the outer edge of the growth cone. It is composed primarily of an actin based cytoskeleton, and contains the lamellipodia and filopodia. Tyr-MTs were probed to visualize the dynamic MTs invading the peripheral domain [38]. We chose to test the effects of 1 pM (10^−12^M), NAP (a concentration affecting tubulin tyrosination in primary astrocytes, Fig. 1e,f) as this concentration was previously shown to be the most effective concentration on axon outgrowth in primary neurons [39]. Our results showed that NAP treatment doubled the average percentage Tyr-MTs covering the peripheral domain area invaded compared to neurons treated with vehicle only (∼10–20% of the ROI, Fig. 5). Fig. 5a shows two representative growth cone structures from a NAP-treated culture with invading Tyr-MTs (red MTs, left side) compared to two control growth cones (right side). Fig. 5b shows a schematic representation of the ROI and the calculated results of 22 growth cones per treatment group is shown in Fig. 5c (p<0.0001). These data suggest that NAP may directly influence MT extension into the peripheral domain of growth cones.

### NAP Increases β3-tubulin Expression in PC12 Cells

**Figure 6:**
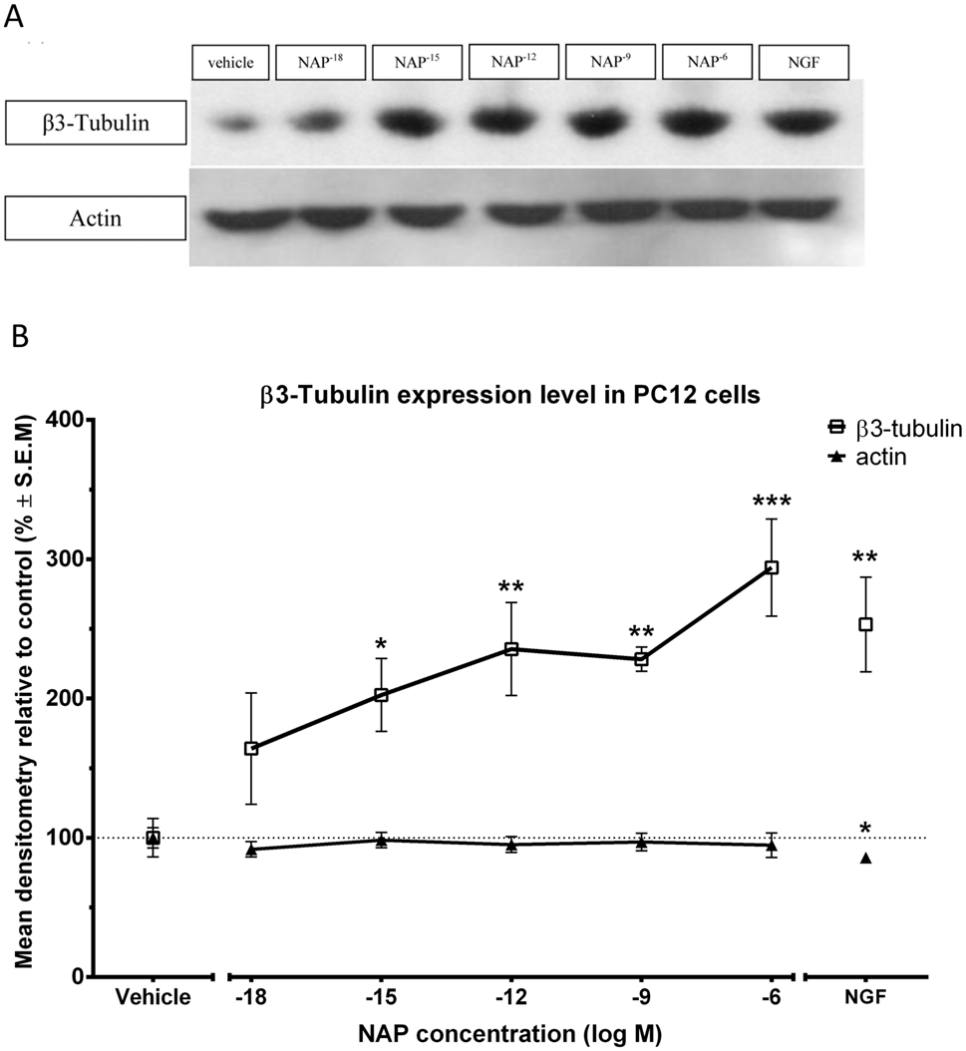
NAP affects β3-tubulin expression levels in PC12 cells. Protein expression levels of β3-tubulin were measured in PC12 cells maintained 8 days in culture. Comparisons were made after exposure to vehicle or different concentrations of NAP (10^−18^M - 10^−6^M). Control cultures were treated with medium only or with NGF (a) Concentration response analysis of protein expression levels were evaluated by western blotting of whole cell extracts with β3-tubulin immunoreactivity (top), and actin, control immunoreactivity (bottom). (b) Densitometric quantification of protein expression performed with ImageJ software. (ANOVA, p = 0.0009, n = 5, post hoc comparison are made in reference to vehicle treatment).

As NAP treatment 1] increased the microtubule network area/cell in PC12 cells (Fig. 4), which precedes neurite outgrowth as well as 2] affected dynamic microtubule invasion into the growth cone in primary neurons (Fig. 5), it was interesting to evaluate whether these changes were associated with specific tubulin markers of neurite outgrowth at a later stage of PC12 differentiation. Previous studies have shown that NAP promotes neurite outgrowth in different primary neuronal cultures including hippocampal, cortical, cerebellar granule neurons and retinal ganglion neurons [40], [41], [42]. β3-tubulin is an established marker for neurite outgrowth, its expression is primarily limited to neurons [5] and purified microtubules enriched in β3-tubulin are considerably more dynamic than those composed from other β-tubulin isotypes [43]. β3-tubulin levels are highest during periods of axon guidance and maturation; decreasing in the adult central nervous system (CNS) but remaining high in the peripheral nervous system (PNS) [44]. Thus, the unique dynamic properties and spatio-temporal expression pattern of β3-tubulin suggest that it could have a specific function for nervous system development and axon maintenance. To ascertain a connection between NAP and neurotrophism, we assessed the effect of long-term NAP treatment on β3-tubulin expression in PC12 cells, in comparison to NGF. PC12 cells were treated with NAP or NGF on alternative days for eight days, and were compared to cells treated with vehicle alone. Cell extracts were analyzed by polyacrylamide gel electrophoresis and western blotting with antibodies specific for β3-tubulin. An increase in the levels of β3-tubulin in response to both differentiation agents was observed (Fig. 6) and the response to NAP exposure was concentration-dependent, with significant increases at concentrations >10^−15^M.

## Discussion

The current set of experiments demonstrates that treatment with the ADNP derived peptide NAP significantly affected the tubulin pool in rat neuronal, neuronal models (PC12) and glial cells in culture. In the neuronal differentiation model, the PC12 cell line, NAP-treatment significantly influenced α tubulin tyrosination cycle which is associated with MT dynamics in the living cell. This effect was coupled to increased MT network area which is directly related to neurite outgrowth. When tested in primary neurons, NAP also affected dynamic Tyr-MT invasion to growth cones. These was associated with neurotrophic effects in PC12 cells, where expression of the MT subunit, β3 tubulin, a marker for neuronal differentiation and neurite outgrowth, increased as a consequence of NAP treatment, similar to NGF treatment effect. The NAP effects on the MT pool were extended to PC12 cell protection against tubulin and tau loss from assembled MT in the cell in the face of toxic environment, high zinc concentrations.

We further showed here that NAP significantly affected the α-tubulin tyrosination cycle in rat primary astrocytes, increasing stable Glu-MT. NAP was previously shown to protect astrocytes from zinc intoxication [21]. Importantly, reduction in stable Glu-MTs is a primary consequence of tau accumulation that initiates mechanisms underlying astrocyte dysfunction and death in human neurodegenerative glial tauopathies [45].

In the PC12 cell model of neuronal differentiation, NAP treatment showed a concentration-dependent effect on the α-tubulin tyrosination cycle, increasing the levels of long living MT, Glu-MT, and also of newly formed MT, Tyr-MT, although to a much lesser extent compared to Glu-MT. The NAP effects were compared to paclitaxel effects, a known MT stabilizing drug, corroborating previously described results of paclitaxel effects at concentrations ≥10^−9^M [25]. Here, we extended the measurements to low concentrations of paclitaxel (below the nanomolar range) that have been shown before to confer neuroprotection [46]. We further extended the measurements to the dynamic Tyr-MT that increased at sub-micromolar paclitaxel concentrations, in agreement with a potential protective effect. As expected, a more extensive concentration-dependent increase in the stable Glu-MT was observed. At very low concentrations, paclitaxel, unlike NAP, did not seem to affect the MT tyrosination cycle. Ratio analysis of Tyr-MT to Glu-MT indicated that NAP treatment resulted in a tendency toward “older”, stabilized MT network. In agreement, NAP treatment increased the levels of cellular polymerized tubulin in a time-dependent manner, as suggested before [21]. When measured under control non-compromising conditions for 2 and 4 hrs. NAP treatment showed an apparent increase of up to 10% (2 hrs.) and a significant increase of ∼15% (4 hrs.) in the tubulin content of the MT pool, as compared to the soluble tubulin pool in PC12 cells.

In contrast to NAP, the paclitaxel effect on the α-tubulin tyrosination cycle increased exponentially (when tested above nanomolar concentrations) and was coupled to a major shift in the MT pool resulting in a significantly higher level of polymerized tubulin (compared to sham controls), even after a 2 hr. incubation period. Thus, unlike NAP, paclitaxel promotes MT assembly and stabilizes MTs by shifting the dynamic equilibrium toward MT assembly, forming extremely stable and non-functional MTs and preventing depolymerisation [47]. Paclitaxel also exhibited a significant effect on NIH 3T3 cells, while NAP had no effect on these cells. This result is in agreement with our previous work [20] in which NAP had no protective effect against severe oxidative stress in NIH3T3 cells and no effect on cell division [48], [49], while paclitaxel inhibits cell proliferation and arrests cell division [50].

Under compromised conditions of zinc intoxication (measured for 4 hrs.) when there is a loss of tubulin and tau from the MT pool, NAP treatment significantly increased the tubulin and tau association with the MT pool, protecting against cell death**.** Previously, NAP was shown to provide protection against zinc intoxication in primary neuronal [20] and in glial cells [21]. While NAP protected against zinc intoxication and increased tau-MT association, paclitaxel treatment (5 µM) seemed to exacerbate the toxicity. This is in-line with published data showing that: 1] Paclitaxel reduces the affinity of tau to tubulin [51]. 2] Paclitaxel displaces tau from MTs [52]. 3] Pre-incubation of tubulin with tau resulted in decreased paclitaxel binding and reduced paclitaxel-induced MT polymerization [53]. 4] High tau expression renders cancer cell resistant to paclitaxel treatment [54]. Thus, the mechanism by which NAP affects the tubulin pool in the cell differs from paclitaxel at the level of tau-MT association.

Furthermore, regarding the association of zinc intoxication with tauopathy, high levels of zinc, up to 1000 µM, can be reached within amyloid plaques in Alzheimer’s disease [55], and there is accumulating evidence indicating that zinc enhances the development of amyloid pathology in Alzheimer’s disease [56], which in turn can induce tau pathology (e.g. [57]). In this respect, a 4 hr. time lapse imaging of tau- Enhanced Green Fluorescent Protein (EGFP) proteins in cells treated with 250 µM zinc showed that the fluorescent MT network disappeared progressively. These previous results suggest that tau-EGFP proteins were detached from MTs and/or that MTs were depolymerized in presence of zinc [28], in agreement with the current data. Taken together, our current results support previous studies that indirectly suggested increased tau association with the MT pool and inhibition of tauopathy in the presence of NAP in vivo [27].

Regarding tau hyperphosphorylation, while previous data from other laboratories suggested a bimodal effect of zinc on tau hyperphosphorylation [28], the currently used conditions did not produce a significant zinc induction of tau hyperphosphrylation. Specifically, zinc intoxication did not result in a significant increase in tau phosphorylation when measured for example with the antibody recognizing p-Ser262 ([58]) or p-Tau^202^ ([59]), (data not shown).

The effect of NAP on the α-tubulin tyrosination cycle coupled with the results of polymerized vs. soluble tubulin at 2 hr. and at 4 hr. incubation periods and further coupled to zinc intoxication, led us to evaluate the effect of NAP on the MT network over an extended time period of 24 hrs. A concentration-dependent increase in mean MT network area/cell in response to NAP treatment was observed. These results suggest that the significant increase in the α-tubulin tyrosination cycle in polymerized MT eventually led to increased protective MT pool (4 hrs.) coupled with increased MT network area/cell (24 hrs.).

Previous observation indicated an effect of NAP on MT organization inside the cells [20], coupled with an ability to promote neurite outgrowth [40], [41], [42], [60] and axonal elongation, all of which requiring stable MT. Paclitaxel on the other hand causes polyneuropathy, mainly sensory, with characteristic features of distal axonal degeneration [61], [62]. In this respect, other studies showed that the protection against axonal degeneration associated with paclitaxel neuropathy is related to relatively lower levels of detyrosinated tubulin, compared to the unprotected state [63]. Furthermore, MT stabilization alone is insufficient to generate cellular processes, since paclitaxel treatment did not alter the overall cell shape, despite the induction of MT bundling within the cell body of Sf9 cells from a moth ovary, while Sf9 tau expressing cells induced processes [64].

Sensory neurons treated with paclitaxel at concentrations above 7×10^−8^M did not elongate extensions. When actively growing neurites are exposed to these levels of paclitaxel, neurite growth stops immediately and does not recommence. The broad processes of neurons cultured for 24 hr. with paclitaxel contain densely packed arrays of MTs that loop back at the ends of the process. In the presence of 7×10^−9^M paclitaxel neurites do grow, although they are broader and less branched compared to control neurites [65]. In contrast, here we show effects of NAP on microtubule invasion into the growth cone.

MT turnover in the growth cone is essential since application paclitaxel to neurons as above [65] or locally to growth cones [66] inhibits neurite growth. One important in vivo observation that was made in studies, with either paclitaxel [67] or epothilone D (another MT stabilizing drug) [68], assessing their potential neuroprotection, is that the respective dose−response curves appeared to be U-shaped, with relatively low doses of the compounds (e.g. 100 times below the cumulative cancer chemotherapeutic dose, in the case of epothilone D) being required for efficacy. Similarly, while 10 nM paclitaxel prevents mutant human-tau-induced swelling of axonal segments, translocation of tau and MT to sub-membrane domains, reduction in the number of MTs along the axon, reversal of the MT polar orientation, impaired organelle transport, accumulation of macro-autophagosomes and lysosomes, compromised neurite morphology and degeneration, higher paclitaxel concentrations (100 nM) do not prevent these events from occurring and in fact facilitate them [69].

NAP short-term effects (tyrosination and MT area/cell and tubulin assembly into MT) showed a bimodal dose response curve in contrast to the sigmoidal dose response curve of MT binding drugs like paclitaxel and colchicine [25]. The bimodal concentration-response curve was also observed in primary cortical neurons that were subjected to tetrodotoxin (TTX) electrical blockade [19], [70]. This may be partially explained by differential protection of neurons and glial cells, different status of cellular differentiation in the tissue culture [21], [71] and different protective epitopes on the NAP primary sequence (NAPVSIPQ) as indicated by systematic alanine amino acid replacement (Ala walk), [72]. Furthermore, a bimodal concentration response curve was noted when measuring axonal length following NAP treatment in cerebellar granule neurons [39].

NAP was shown to promote neurite outgrowth in different primary neuronal cultures including hippocampal, cortical, cerebellar granule neurons and retinal ganglion neurons [39], [40], [41], [42], [60]. β3-tubulin, an established marker for neurite outgrowth with expression primarily limited to neurons [5] plays a role in early neuritogenesis, concomitantly or in coordination with other MT associated proteins (MAPs) [6], enhancing MT polymerization [7].

As introduced above, purified MTs enriched in β3-tubulin are considerably more dynamic than those composed of other β-tubulin isotypes [43]. MTs with different isotype composition have different functions [2], [3] and display different dynamic properties [43]. In terms of neuroprotection, the expression of β3-tubulin renders the MTs less sensitive to oxidative damage [73], [74]. It has been shown that β3-tubulin is conditionally expressed as an adaptive mechanism of resistance to a stressing microenvironment featuring oxygen-poor conditions and low nutrient supply [75] unraveling a functional connection between β3-tubulin expression and cell survival. Importantly, mutations in the gene encoding β3-tubulin (TUBB3) cause an increase in Glu-MT with increased MT stability [76] and see below. Modifications like detyrosination, are regulated by specific enzymes, and each can profoundly impact the capacity of the MT to interact with other proteins [12], [77], [78], [79], [80].

Here, we showed that NAP treatment increased the MT network area/cell, which precedes neurite outgrowth. We also showed that long-term treatment with NAP increased β3-tubulin expression, a marker for neurite outgrowth, neurodifferentiation and neuroprotection, providing a time course and an initial mechanistic background for the observed biological action.

When addressing the NAP effect of neurotrophism/neuroprotection, a previous study associated mutations in human β3-tubulin in perturbation of MT dynamics (increases in de-tyrosinated tubulin), kinesin interactions, and axon guidance [76], which are linked to a spectrum of human nervous system disorders that are now called the TUBB3 syndromes. Our original studies related NAP activity with β3-tubulin expression, which is not found in NIH3T3 [20]. In this respect, Sudo and Baas demonstrated that katanin severing of MT can lead to microtubule breakdown in axons. They have further shown that this process is accentuated by tau silencing and is inhibited to a large extent by NAP treatment. In contrast to axons, in minor processes, tau silencing was not required for microtubule severing by katanin, and NAP provided protection in the absence and in the presence of tau silencing. Furthermore, Sudo and Baas also showed that NAP protected against katanin – induced microtubule breakdown in RFL-6 fibroblasts only when β3-tubulin was ectopically expressed [81].

The basal level of β3-tubulin expression or other neuronal differentiation related proteins may explain the different dose response curves observed for NAP treatment in non-differentiated and differentiated PC12 cells and the dose response curves obtained after long treatment of NAP. In this manner, differences can also be found in the tubulin required protein components in cell protection vs. neurite outgrowth [2], [3]. Furthermore, specific MT associated proteins contributing to MT function may play a part in NAP action. Thus, Chen and Charness have shown that the NAP mechanism on axon growth requires Fyn kinase [39] which interacts with tau [82], while in vivo, NAP protected against the microtubule associated protein MAP6 (STOP) deficiencies [83]. Taken together, these studies suggest that NAP repairs the severed MT system, depending on the availability of MAPs and the primary composition of the MT backbone.

We have previously discovered a NAP-dependent reduction in activated glycogen synthase kinase-3-β (GSK3β) that is associated with the pathological hyperphosphorylation of tau, and the formation of neurofibrillary tangles [18]. The NGF-related cascade of neurite outgrowth includes inhibition/inactivation of GSK3β [84], [85], coupled to increases in β3-tubulin and MT polymerization/dynamics.

In contrast to the similar effects found on axonal/neurite outgrowth of NAP and NGF, our previous studies did not show similarities in neuroprotection against TTX. While NAP provided protection, NGF (as well as other neurotrophins) did not, at concentrations that are active in other systems [86]. Furthermore, in PC12 cells expressing myotonic dystrophy type 1-associated CTG repeats, NGF treatment resulted in reduction in expression of the MT associated proteins MAP6/STOP, while NAP protects against STOP – associated deficiencies [83]. The neurotrophic activity of either NAP or NGF were also associated with polyADP ribosylation in vitro [60], [87] and polyADP ribosylation was in turn associated with plasticity and memory formation [88].

The neuroprotective effect of NAP paralleled protection against apoptosis (cytochrome-c release), protection against caspase 3 activation [59] and MT breakdown protection [89], [90]. Recent studies suggested that MT polymerization re-establishment by protective paclitaxel concentrations reduced amyloid β oligomers [91], which in turn have been associated with the formation of hyperphosphorylated tau [57]. Thus, protection of MT polymerization by NAP has far reaching mechanistic consequences on protection in Alzheimer’s disease and related tauopathies. It is our working hypothesis that by subtle changes to the MT network paralleled by effects on key enzymes/pathways, NAP confers MT related neuroprotection.

The current studies provide additional weight to observed effects of NAP on tau-related pathology and MT dysfunction [18], [27], [57], [92], [93]. Thus, NAP effects on the MT structures and associated impact on MT pool and tau recruitment to MT may provide an explanation for the resulting neuroprotection. NAP (davunetide) is in phase 2/3 clinical trial in progressive supranuclear palsy (PSP) a disease presenting MT deficiency, tau pathology with tangles sharing epitopes with tyrosinated and detyrosinated tubulin [94]. Understanding of the NAP cellular mechanism of action as a first in class investigational compound paves the path to the discovery of novel ways to combat devastating dementias.
